# Boosting effect of regular sport practice in young adults: Preliminary results on cognitive and emotional abilities

**DOI:** 10.3389/fpsyg.2022.957281

**Published:** 2022-09-28

**Authors:** Noemi Passarello, Ludovica Varini, Marianna Liparoti, Emahnuel Troisi Lopez, Pierpaolo Sorrentino, Fabio Alivernini, Onofrio Gigliotta, Fabio Lucidi, Laura Mandolesi

**Affiliations:** ^1^Department of Humanities, “Federico II” University of Naples, Naples, Italy; ^2^Department of Social and Developmental Psychology, “Sapienza” University of Rome, Rome, Italy; ^3^Department of Motor Sciences and Wellness, University of Naples “Parthenope”, Naples, Italy; ^4^Institut de Neuroscience des Systemès, Aix-Marseille University, Marseille, France

**Keywords:** sport, cognition, emotion regulation, physical activity, alexithymia

## Abstract

Several studies have shown that physical exercise (PE) improves behavior and cognitive functioning, reducing the risk of various neurological diseases, protecting the brain from the detrimental effects of aging, facilitating body recovery after injuries, and enhancing self-efficacy and self-esteem. Emotion processing and regulation abilities are also widely acknowledged to be key to success in sports. In this study, we aim to prove that regular participation in sports enhances cognitive and emotional functioning in healthy individuals. A sample of 60 students (mean age = 22.12; SD = 2.40; M = 30), divided into sportive and sedentary, were subjected to a neuropsychological tests battery to assess their overall cognitive abilities (Raven's Advanced Progressive Matrices, APM), verbal and graphic fluency (Word Fluency Task and modified Five Point Test, m-FPT), as well as their emotional awareness skills (Toronto Alexithymia Scale, TAS-20). Our results showed that sportive students performed better than sedentary ones in all cognitive tasks. Regarding emotional processing abilities, significant differences were found in the TAS-20 total score as well as in the Difficulty Describing Feelings (DDF) subscale and the Difficulty Identifying Feeling (DIF) subscale. Lastly, gender differences were found in the External-Oriented Thinking (EOT) subscale. Overall, our findings evidence that PE has positive effects on cognitive functioning and emotion regulation, suggesting how sports practice can promote mental health and wellbeing.

## Introduction

Physical exercise (PE) is defined as a form of physical activity that is planned, structured, and repetition-based, with the goal of improving or maintaining individual components of physical fitness. PE is defined as a sports activity when an individual systematically trains under expert supervision for a specific technical gesture (Mandolesi et al., 2018).

A substantial body of evidence has shown that PE induces a brain plasticity phenomenon (Fernandes et al., 2017; Mandolesi et al., 2018) that represents the ability of the brain to reorganize and change after proper stimulation, thus improving its performance (Serra et al., 2011; Mandolesi et al., 2017; Gelfo et al., 2018).

For a few years now, the beneficial effects of PE on mood and cognition across the life span have become a topic of interest. Much evidence suggests that PE improves behavior and cognitive functioning, reducing the risk of various neurological diseases, protecting the brain from the detrimental effects of aging, facilitating body recovery after injuries, and enhancing self-efficacy and self-esteem (Lista and Sorrentino, 2010; Basso and Suzuki, 2017). Cross-sectional studies have shown that PE boosts memory abilities, attentional processes' efficiency, and executive functioning (Colcombe and Kramer, 2003; Grego et al., 2005; Pereira et al., 2007; Chieffi et al., 2017; Erickson et al., 2019). Specifically, executive functions are frequently targeted for PE studies, as they play a crucial role in both mental and physical health (Davis et al., 2011), success in school, and quality of life (Bailey, 2007), as well as social, cognitive, and psychological development (Diamond, 2016). Nonetheless, inhibition control, working memory, and cognitive flexibility are considered to be key abilities for sports practice (Ludyga et al., 2016).

Emotion processing and regulation skills are also widely acknowledged to be key to success in sports (Friesen et al., 2013). The ability to process and regulate emotions successfully is regarded by many as an important psychological skill in sportive individuals (Jodat et al., 2015). Several studies demonstrate the enormous benefits of structured and constant sports training in terms of improving motivation and emotional wellbeing (Ghasempour et al., 2013; Behroozi and Abdimoghadam, 2014). The ability to successfully process and regulate our emotions requires emotional awareness. In some individuals, this ability is deficient, resulting in difficulties identifying, describing, and analyzing what they are experiencing emotionally (Passarello et al., 2021). Considering the importance of emotional regulation in everyday life, constant and structured PE seems to be a protective factor for this difficulty (Jodat et al., 2015). In spite of this, there are still few studies that examine the correlation between sport and emotional regulation.

In this study, we aim to demonstrate that regular participation in sports can lead to an improvement in cognitive and emotional functioning. To do this, a sample of 60 students (mean age = 22.12, SD = 2.40), divided into sportive and sedentary, were subjected to a neuropsychological tests battery to assess their overall cognitive abilities, and verbal and graphic fluency (Raven Advanced Progressive Matrices, APM; World fluency test; modified-Five Points Test, m-FPT), as well as their emotional awareness skills (Toronto Alexithymia Scale, TAS-20). We hypothesized that sportive students would demonstrate enhanced cognitive abilities, along with better skills at recognizing and describing their emotions, compared to sedentary students.

## Materials and methods

### Participants

We tested 60 students from the University of Naples “Federico II.” A survey was conducted to ask students if they practice any type of sport, how long they had been practicing it, how often, and at what intensity. From the output of this survey, we selected our sample, and we sorted the students into two groups as follows: Sportive = 15 females (mean = 22; SD = 2.76) and 15 males (mean = 22.18; SD = 3.15); Sedentary = 15 females (mean = 23; SD = 2.04) and 15 males (mean = 21.30; SD = 1.26). Participants who had practiced sports for at least 2 years with a frequency of 3 times a week and with a moderate level of intensity belonged to the sportive group. All participants were in good health. Inclusion criteria were normal or corrected-to-normal vision and right-handedness. Alternatively, exclusion criteria comprised the current or past presence of psychopathology, psychiatric, neurological, or motor disorders, or other medical illness. The participants were voluntarily enrolled after written informed consent was obtained. The study was approved by the Local Ethics Committee of the University of Naples “Federico II” (protocol number: 12/2020) and was carried out in accordance with the Declaration of Helsinki.

### Neuropsychological assessment

To evaluate the typical development of all the participants, Raven's Advanced Progressive Matrices (APM) (Raven, 1962) were administered. Specifically, we used set 1 consisting of the first 12 matrices. To complete each item, one must replace the missing part of a model, according to a criterion of increasing difficulty. Model figures consist of graphic patterns that change from left to right and from top to bottom; the subject must understand the underlying logic and apply it in order to come up with the solution. APM total score allowed us to assess abstract reasoning and global cognitive functioning.

Verbal fluency was also assessed through word fluency test (Carlesimo et al., 1996). In this test, participants were asked to produce as many words as possible, beginning with specified letters of the alphabet (A; F; S), in a limited time interval of 60 s. Total word produce, word errors (i.e., word beginning with a different letter), and word repetitions were analyzed.

The five-point test (FPT) is a highly reliable nonverbal measure of executive functioning, assessing the graphic-figural fluency of participants (Fernandez et al., 2009). In fact, it measures the ability of an individual to produce geometric drawings or unique figures within a given time interval (3 min) (Cattelani et al., 2011). The modified FTP version (m-FTP) consists of an A4 sheet with 40 square matrices, each containing five dots; four of them are placed at the vertices and one is placed in the middle. Participants must connect two or more dots in each square with straight lines. Additionally, they must not repeat the same shape two times and must not draw lines that do not connect the dots. Through this, we can examine three subdomains of executive functions, namely, flexibility, rule breaking, and strategic performance. The following parameters were evaluated: (A) total drawings: number of total drawings made in 3 min; (B) drawings with errors: number of drawings breaking the rules and/or repeating previously drawn shapes; (C) error index: number of drawings with errors divided by the number of total drawings multiplied by 100; (D) number of unique drawings: calculated by subtracting the number of drawings with errors from the number of total drawings; and E) strategy index: number of drawings with strategy divided by number of unique drawings. All the neuropsychological instruments used are validated for the Italian language and culture (Di Fabio and Clarotti, 2007; Carlesimo et al., 1996; Cattelani et al., 2011).

### Toronto alexithymia scale (TAS-20)

The TAS-20 (Bagby et al., 1994) is a 20-item self-report test considered to be the most reliable self-assessment questionnaire for measuring alexithymia, an affective-cognitive disorder characterized by difficulty in identifying and describing own emotions and in being interested in understanding those of others (Nemiah and Sifneos, 1970). TAS-20 consists of three subscales, each representing one aspect of alexithymia: the Difficulty Describing Feelings (DDF) subscale consisted of five items (2, 4, 11, 12, and 17); the Difficulty Identifying Feeling (DIF) subscale consisted of seven items (1, 3, 6, 7, 9, 13, and 14); and the External-Oriented Thinking (EOT) subscale, measuring the tendency of individuals to focus their attention externally, consisted of eight items (5, 8, 10, 15, 16, 18, 19, and 20).

Participants respond to TAS-20 using a 5-point Likert scale, where 1 indicates strongly disagree and 5 indicates strongly agree. Items 4, 5, 10, 18, and 19 are negatively keyed. Alexithymia scores are determined by summing responses to all 20 items, while scores for each of the subscales are determined by summing the responses in each scale. Scores of 61 or higher are considered alexithymic; scores of 50 or lower are considered non-alexithymic, with a borderline between 50 and 60. In this study, we used the Italian standardized and adapted version of the TAS-20 (Bressi et al., 1996).

### Statistical analyses

All statistical analyses were conducted with the JASP 0.16.1.0 software. The 2 × 2 ANOVA was used to analyze the effect of gender (males, females) and sport practice (sportive, sedentary) on neuropsychological tests (APM, word fluency, and m-FPT) and TAS-20. *p* ≤ 0.05 were considered statistically significant. *Post-hoc* comparisons were conducted through Tukey's test.

## Results

### Neuropsychological assessment

**Figure 1:**
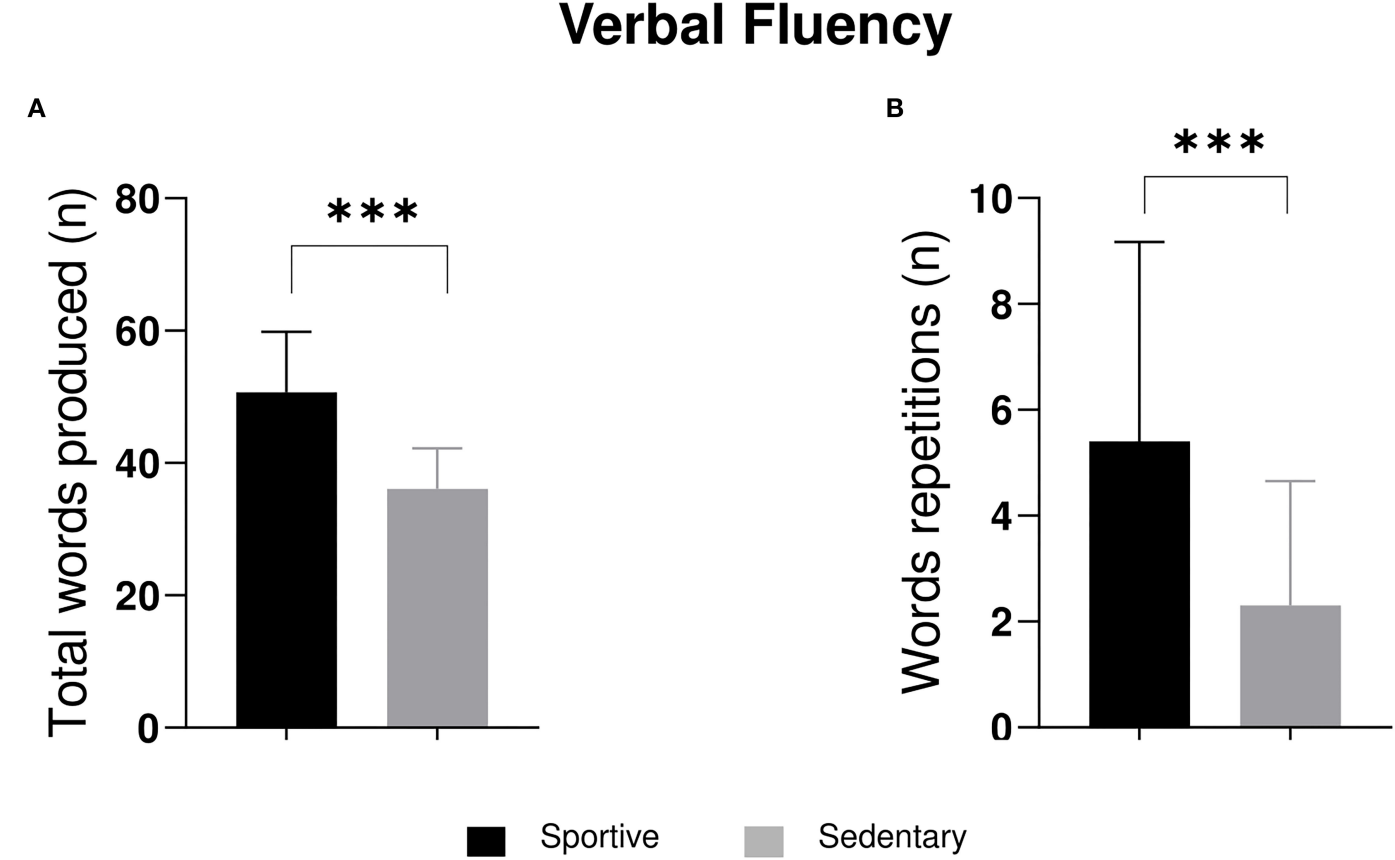
The 2 × 2 ANOVA significant outputs on **(A)** total words produced and **(B)** words repetitions. ***p ≤ 0.001.

**Figure 2:**
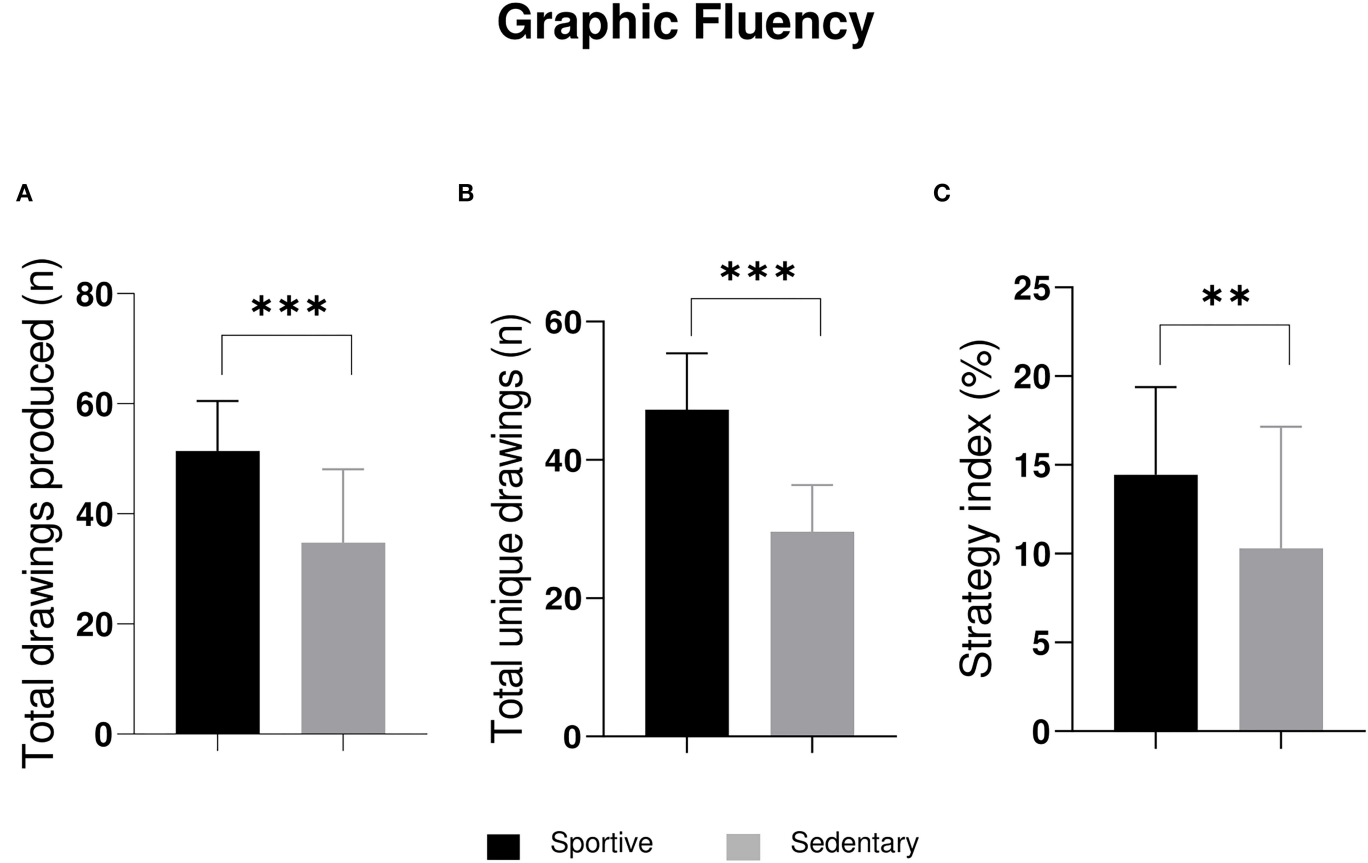
The 2 × 2 ANOVA significant outputs on **(A)** total drawings produced, **(B)** total unique drawings, and **(C)** strategy index. **p ≤ 0.01; ***p ≤ 0.001.

The 2 × 2 ANOVA outputs on APM are shown in Table 1, while word fluency and m-FPT outputs are shown in Figures 1, 2.

**Table 1:** The 2 × 2 ANOVA outputs on advanced progressive matrices (APM).

**Factors**	**F_1, 56_**	***p***	**p _Tukey_**
Sport	5.13	0.02*	0.02*
Sex	0.01	0.90	0.90
Sport*Sex	1.25	0.28	0.28

^*^p ≤ 0.05.

Significant differences were found in APM score (*F*_1, 56_ = 5.14; *p* = 0.03), word production (*F*_1, 56_ = 52.62; *p* ≤ 0.001), and word repetition (*F*_1, 56_ = 14.60; *p* ≤ 0.001) for sport factor. *Post-hoc* comparisons revealed significant difference between sportive students and sedentary students in APM score (mean sportive = 10.80; mean sedentary = 10.16), word production (mean sportive = 50.70; mean sedentary = 36.07), and word repetition (mean sportive = 5.40; mean sedentary = 2.30). As for word errors, we found significant differences for sex factor (*F*_1, 56_ = 6.20; *p* = 0.02). *Post-hoc* comparisons on word errors revealed significant differences between males and females (mean males = 1.43; mean females =0.77).

Several results were found among m-FPT parameters. The 2 × 2 ANOVA revealed significant differences in the total drawing for sport factor (*F*_1, 56_ = 31.22; *p* ≤ 0.001) (Figure 2A). *Post-hoc* comparisons revealed a significant difference between sportive students and sedentary students in total drawings productions (mean sportive = 51.43; mean sedentary = 34.77). No significant differences were found for sex (*F*_1, 56_ = 0.36; *p* = 0.55) or interception (*F*_1, 56_ = 0.29; *p* = 0.59).

Moreover, significant differences were found in total unique drawings for the sport factor (F_1, 56_ = 82.30; *p* ≤ 0.001) (Figure 2B). *Post-hoc* comparisons revealed a significant difference between sportive students and sedentary students in unique drawings productions (mean sportive = 47.30; mean sedentary = 29.60). No significant differences were found for sex (*F*_1, 56_ = 0.49; *p* = 0.49) or interception (*F* = 0.25; *p* = 0.62). No significant differences were found in the error index for sports, sex, or interception.

Finally, significant differences were found in strategy for sport factor (*F*_1, 56_ = 7.15; *p* = 0.01) (Figure 2C). *Post-hoc* comparisons revealed a significant difference between sportive students and sedentary students in strategy use for drawing (mean sportive = 0.14; mean sedentary = 0.10). No significant differences were found for sex (*F*_1, 56_ = 0.19; *p* = 0.66) or interception (*F*_1, 56_ = 1.66; *p* = 0.20).

### TAS-20

Among our 60 participants, only two were found to be alexithymic with an average score of 65. The rest of the group obtained an average score of 44.93. Twenty of the remaining participants obtained an overall score that falls in the critical range of 50–60 (Supplementary Table 1).

**Figure 3:**
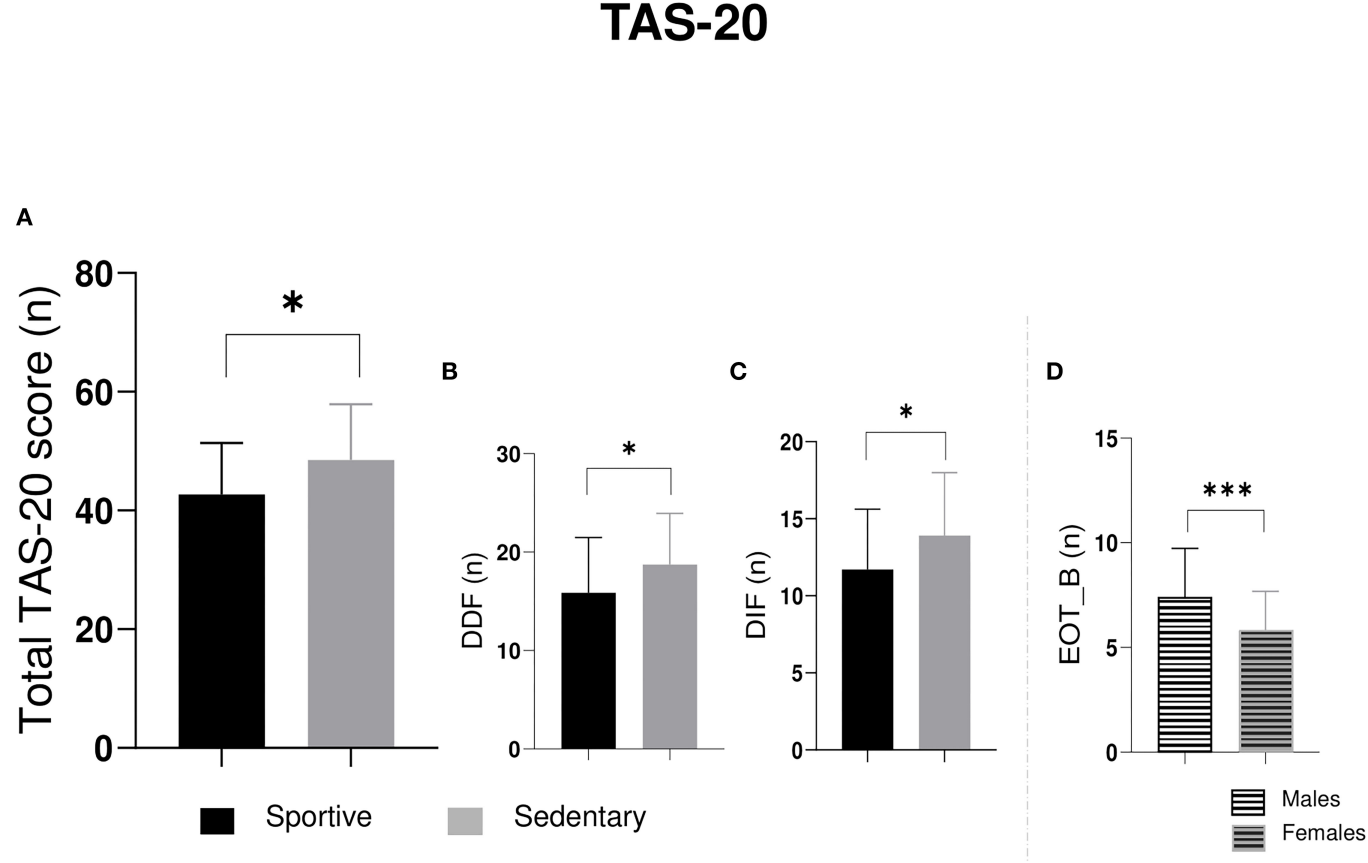
The 2 × 2 ANOVA significant outputs on **(A)** total TAS-20 score, **(B)** DIF factor, **(C)** DDF factor, and **(D)** EOT_B factor. DIF, difficulty identifying feelings; DDF, difficulty describing feelings; EOT, external-oriented thinking. *p ≤ 0.05; ***p ≤ 0.001.

As for TAS total score, the 2 × 2 ANOVA revealed a significant difference for sport factor (*F*_1, 56_ = 6.01; *p* = 0.02) (Figure 3A). *Post-hoc* comparisons lower alexithymic score in sportive students rather than sedentary students (mean sportive = 42.70; mean sedentary = 48.50). No significant differences were found for sex (*F*_1, 56_ = 0.18; *p* = 0.67) or interception (*F*_1, 56_ = 0.26; *p* = 0.61).

TAS factor DIF was significantly different for sport factor (*F*_1, 56_ = 4.52; *p* = 0.04) (Figure 3B). *Post-hoc* comparisons revealed minor difficulties in identifying feelings in sportive students rather than sedentary students in total score (mean sportive = 11.70; mean sedentary = 13.90). No significant differences were found for sex (*F*_1, 56_ = 2.20; *p* = 0.14) or interception (*F*_1, 56_ = 1.07; *p* = 0.80).

TAS factor DDF was also significantly different for sport factor (*F*_1, 56_ = 4.37; *p* = 0.04) (Figure 3C). *Post-hoc* comparisons revealed minor difficulties in describing feelings in sportive students rather than sedentary ones (mean sportive = 15.87; mean sedentary = 18.73). No significant differences were found for sex (*F*_1, 56_ = 48.60; *p* = 0.19) or interception (*F*_1, 56_ = 72.60; *p* = 0.11). No significant differences were found in EOT_A for sport, sex, and interception.

Finally, TAS factor EOT_B was significantly different for sex factor (*F*_1, 56_ = 7.78; *p* = 0.007) (Figure 3D). *Post-hoc* comparisons revealed more external-oriented thinking in males rather than females (mean males = 7.37; mean sedentary = 5.80). No significant differences were found for sport (*F*_1, 56_= 0.09; *p* = 0.77) or interception (*F*_1, 56_ = 0.08; *p* = 0.76).

## Discussion

In accordance with our hypotheses, our results showed that sportive students performed better than sedentary ones on tasks of global intelligence, verbal and graphic fluency, and emotional awareness. In spite of our sample consisting of students with typical development, as evidenced by the normal Raven matrices (APM) scores, we found that the sportive group performed better than the sedentary group in APM. Several studies showed that both children and young adults' experiences in sport and PE contribute to the mental acuity, skills, and strategies that are important for navigating challenges faced across the life span (Donnelly et al., 2016). Neuroscience advances have enabled significant progress in linking PE to cognitive performance, as well as to brain structure and function (Mandolesi e al., 2018). It was found that PE could determine structural changes in gray matter volume in frontal and hippocampal regions and enhance the release of neurotrophic factors, such as peripheral BDNF (Serra et al., 2011; Mandolesi et al., 2018). These effects are reflected on cognitive functioning. In our study, sportive students performed better in word fluency task, producing more words than sedentary students. Further, aerobic exercise improves verbal fluency among healthy elderly (Bullo et al., 2015). This evidence validates the selective improvement hypothesis, which states that aerobic PE selectively improves brain activities associated with the frontal and prefrontal regions (Weinstein et al., 2012; Nocera et al., 2015, 2020). Indeed, PE is associated with high scores on tests that evaluate executive functioning (Verburgh et al., 2014; de Greeff et al., 2018; Xue et al., 2019; Serra et al., 2021). In our study, it is important to note that the m-FPT provides insight into executive functioning (Weinstein et al., 2012), and also in this test, we found significant differences in favor of sports students. It is important to note that, among our sample of sportive students, more repetitions of words were also recorded. However, the increased use of repetition by sportive students appears to be in contrast to the results discussed so far; this was only true for verbal fluency and not for graphic fluency. Sportive students were in fact more productive than the sedentary ones in the m-FPT, but more importantly, they produced more unique designs and used strategies more often. This dissociation between verbal and visuospatial abilities (including graphic fluency competencies) has long been associated with a hemispheric lateralization (Lardone et al., 2021). Several studies have shown that performance in verbal fluency task was associated with increased left prefrontal activity, whereas performance in visuospatial fluency task was associated with increased bilateral prefrontal activity (Marin et al., 2017; Cipolotti et al., 2020). An interesting work (Elfgren and Risberg, 1998) takes also in account the cognitive strategy used during fluency tasks. Participants were asked to explain the type of strategy they used to solve both verbal and visuospatial task: they could have used a purely verbal strategy (i.e., think about nameable objects), a purely visual strategy (i.e., use visual imagery), or a mix of the two. In the visuospatial fluency task, participants used more mixed strategies, whereas in the verbal fluency task, they used mixed and verbal strategies. Both in visuospatial and verbal fluency tasks, the application of mixed strategies was associated with increased bilateral activity in the prefrontal cortex. Meanwhile, the application of verbal strategies only was associated with an increase in activity just in the left dorsolateral prefrontal cortex. We can speculate that PE enhances hemispheric lateralization, improving both verbal skills linked to left-hemisphere activity and visuospatial skills that appear to be linked to bilateral activity (Wang et al., 2016). However, it does not have a specific effect on the left-hemisphere-related activity required to improve the use of appropriate strategies to solve verbal fluency tasks. Although more results are needed to confirm the latter hypothesis, we can conclude that regular practice of a sport improves overall cognitive functioning. Additionally, our results showed that sportive students were more aware of their own emotions than sedentary ones, displaying fewer difficulties in identifying and describing their own feelings. PE beneficial effects on emotional processing has been widely reported. Epidemiological studies have shown PE soothing effects on depression (Mammen and Faulkner, 2013) and anxiety (Knapen et al., 2009). Numerous studies have shown that most aerobic exercises, regardless of their type, lead to an enhanced positive mood afterward (Fernandes et al., 2017).

Alexithymia, or “no words for feelings,” is a personality trait that is associated with difficulties in emotion recognition and regulation (Swart et al., 2009). In sport, it is mostly associated with disorders, such as anxiety, depression, overtraining (burnout), addiction, and risky sports behavior (Woodman et al., 2009). However, some studies have proved that sport practice can improve emotional recognition and regulation, and accordingly, fewer alexithymic traits can be found in sportive individuals (18). These data agree with our result in which sportive students have lower scores on TAS-20 (Figure 3A). It has been reported that sport practice had several benefits for students, including less difficulty in “identifying and distinguishing physical feelings and sensations,” better sleep, and higher scores on happiness and life satisfaction (Manfredi, 2022). Sportive students also showed more external-oriented thinking than sedentary ones. The results of our work do not directly confirm this finding, but they do detect a gender difference regarding factor 3B of the TAS-20 (external-oriented thinking). Specifically, males tend to use more external-oriented thinking than females, regardless of whether they practice sport. Based on this finding, it may explain why some scientific evidence suggests that alexithymia is associated with increased athletic activity, especially in high-risk sports. Although it is true that there is a greater prevalence of alexithymia in high-risk sports contexts (Woodman et al., 2009; Woodman and Welch, 2021), these data may also be biased due to gender. It is possible that the gender differences would be marked mainly in the verbalization of emotions and in external-oriented thinking, since both of these dimensions of alexythimia are more frequently associated with males, due to social desirability bias (Proença Lopes et al., 2022a,b). Since our sample was balanced by gender, we can speculate that sportive individuals are less alexithymic, and instead, the higher prevalence of alexithymia in high-risk sporting contexts is probably due to gender; however, more evidence is needed.

The results from our study agree with the majority of studies on PE and its effect on psychological wellbeing (Ghasempour et al., 2013). As pointed out by these studies, while practicing a sport, individuals not only acquire physical growth but also experience personal, mental, and emotional growth as well. Moreover, by creating a context for increasing their sense of empowerment and self-worth, sport's environment prevents young adults to develop psychological, emotional, and affective problems (e.g., alexithymia and its components). Sport practice can also improve positive attitudes and change negative beliefs and dysfunctional views of people's lives and their surroundings (Cox, 2012). To get the full picture, we also need to assess some socio-psychological factors that can contribute to the cognitive functioning differences between those who practice sports and those who lead a sedentary lifestyle. Indeed, the characteristics of sportive populations often include a favorable economic condition along with goal-directed behavior or proactivity that differ from those of sedentary ones (Hallmann and Breuer, 2014; Lucia et al., 2021). While these socio-economic factors have not been examined in this preliminary study, they provide a reflection for future research which will allow us to study the specific weights of each psychological factor involved in defining a specific “cognitive sportive profile.” There are, however, other limitations to our results, making them even more preliminary. A larger sample is needed to further strengthen the association between sport practice and psychological wellbeing, which also allows us to consider individual and gender-related differences. It should be noted that most of our sample was composed of amateur tennis players who belonged to the same sports association. Since we know that different types of physical activity have different effects on our cognitive abilities, it would be worthwhile to investigate the effects of other sports disciplines. According to Mandolesi et al. (2018), the benefits of PE on cognitive functioning and wellbeing are different depending on whether the activity is performed aerobically or anaerobically. Moreover, there is a major difference between chronic and acute PE, with chronic aerobic exercise being mostly associated with structural and functional neuroplastic changes along with enhanced cognitive functions (Colcombe et al., 2006; Mandolesi et al., 2017). Finally, our study was conducted following COVID-19 lockdown during which our students continued to train and to resume outdoor physical activity once possible. It could be useful to know whether PE's beneficial effects are sustained if physical activity is interrupted for an extended period.

In conclusion, our preliminary results show that regular exercise and participation in sport can positively affect cognitive and emotional functioning. Our sportive students performed better than sedentary students in terms of global intelligence, graphic and verbal fluency, as well as recognizing and describing emotions. It can be concluded that the promotion of PE in young and at-risk populations is an important priority for the promotion of psychological wellbeing, considering its beneficial function.

## Data availability statement

The raw data supporting the conclusions of this article will be made available by the authors, without undue reservation.

## Ethics statement

The studies involving human participants were reviewed and approved by Local Ethics Committee of the University of Naples Federico II (protocol number: 12/2020). The patients/participants provided their written informed consent to participate in this study.

## Author contributions

NP, LV, and ML performed the research. NP, ET, and PS analyzed the data. NP, FA, OG, FL, and LM wrote the manuscript. All authors designed the research, read, revised, and approved the final manuscript.

## Funding

This research was supported by funding from the Department of Humanities, University of Naples Federico II (Fondi ricerca dipartimentale 70%, 2020 and 2021) to LM.

## Conflict of interest

The authors declare that the research was conducted in the absence of any commercial or financial relationships that could be construed as a potential conflict of interest.

## Publisher's note

All claims expressed in this article are solely those of the authors and do not necessarily represent those of their affiliated organizations, or those of the publisher, the editors and the reviewers. Any product that may be evaluated in this article, or claim that may be made by its manufacturer, is not guaranteed or endorsed by the publisher.
