# Supplementary material for: Boosting effect of regular sport practice in young adults: Preliminary results on cognitive and emotional abilities
Source: Front Psychol. 2022 Sep 28;13:957281. doi: 10.3389/fpsyg.2022.957281 (PMC9555280; doi:10.3389/fpsyg.2022.957281)
Supplement: Supplementary file 1 [file Table_1.docx]

Supplementary Material

# Supplementary Figures and Tables

**Table S1.** Number of participants with high, borderline, and low alexithymic traits, divided between sportive and sedentary. High-alexithymic: scores higher than/ equal to 61; Bordeline-alexithymic scores of between 60 and 5; Low-alexithymic: scores below 51.

|  | High-Alexithymic | Borderline-Alexithymic | Low-Alexithymic |
| --- | --- | --- | --- |
| Sportive | 0 | 6 | 16 |
| Sedentary | 2 | 12 | 24 |
